# Mitochondria transfer in neurological disorders: the key role of neuroglia

**DOI:** 10.1186/s13024-026-00953-1

**Published:** 2026-05-21

**Authors:** Jie Jiao, Hui Chen, Alexei Verkhratsky, Yixun Su, Chenju Yi

**Affiliations:** 1 Department of Geriatrics, Seventh Affiliated Hospital of Sun Yat-sen University, No. 628 Zhenyuan Road, Guangming District, Shenzhen, 518107 China; 2 School of Life Sciences, Faculty of Science, University of Technology Sydney, Ultimo, NSW 2007 Australia; 3 Faculty of Biology, Medicine and Health, The University of Manchester, Manchester, UK; 4 International Joint Research Centre on Purinergic Signalling of Sichuan, Province Chengdu University of Traditional Chinese Medicine, Chengdu, China; 5 Celica, BIOMEDICAL, Tehnološki park 24, Ljubljana, 1000 Slovenia; 6 Department of Forensic Analytical Toxicology, School of Forensic Medicine, China Medical University, Shenyang, China; 7 Guangdong Provincial Key Laboratory of Digestive Cancer Research, The Seventh Affiliated Hospital of Sun Yat-sen University, Shenzhen, 518107 China; 8 Shenzhen Key Laboratory of Chinese Medicine Active Substance Screening and Translational Research, Shenzhen, 518107 China

**Keywords:** Neuroglia, Tunnelling nanotubes, Extracellular vesicles, Neurodegeneration, Traumatic brain injury, Ageing, Mitochondrial transplantation

## Abstract

Mitochondria transfer has emerged as a distinctive mechanism for intercellular communication and neuronal homeostasis. Neurones, owing to their unique bioenergetic demands, are particularly vulnerable to mitochondrial dysfunction, a shared pathogenetic feature across many neurological conditions, including neurodegenerative disorders, cerebrovascular diseases, and brain injuries. Intercellular transfer of mitochondria represents a potential adaptive mechanism rectifying compromised mitochondrial function. Neuroglial cells, especially astrocytes and microglia, frequently act as mitochondrial donors, supplying functional mitochondria to stressed neurones to restore bioenergetic capacity and influence disease trajectories. However, mitochondria transfer is intrinsically context dependent and can exert opposing effects. In addition to providing metabolic support, damaged mitochondria may also be transferred, propagating pathological signals, and exacerbating tissue injury. Moreover, in advanced disease states, mitochondrial malfunction often affects all cell types in the nervous system, including neuroglia, limiting the availability of healthy endogenous mitochondrial donors. This review critically examines mitochondria transfer in neurological diseases, with a focus on glial contribution and underlying mechanisms, and outlines key challenges and opportunities for advancing both mechanistic understanding and therapeutic translation.

## Introduction

Mitochondria, the powerhouse of eukaryotic cells, are essential to support ATP production, substrate metabolism, Ca^2+^ homeostasis, and the regulation of apoptotic signalling [1]. The discovery of transcellular mitochondria transfer in 2006 [2] revised the long-held view of mitochondria as cell-anchored organelles, demonstrating that mitochondria can move between cells and restore energetics of metabolically compromised recipient cells [3]. This phenomenon is of particular relevance to the nervous system, which is highly dependent on mitochondrial oxidative phosphorylation for ATP production to meet its substantial energy demand. Indeed, the adult brain consumes ~ 20–25% of total body glucose intake despite constituting only 2% of body mass [4]. This metabolic demand is even greater during development, because the developing brain requires up to 50% of the total body glucose supply [5]. The post-mitotic nature of mature neurones precludes the dilution or removal of damaged mitochondria through cell division, rendering them particularly vulnerable to the cumulative effects of mitochondrial malfunction [6]. At the same time, the complex cellular architecture of nervous tissue, characterised by extensive neuron-glia networks, creates multiple potential pathways for intercellular mitochondrial exchange [7].

In the central nervous system (CNS), neuroglial cells appear as the primary donors of healthy mitochondria and acceptors of damaged mitochondria transferred from the neurones. Astrocytes possess elaborate terminal processes known as leaflets closely contacting neuronal somata and synapses [8, 9], these leaflets are strategically positioned to support neuronal homeostatic and metabolic needs [10]. Microglia are the resident immune cells of the brain endowed with multiple physiological functions [11, 12], responding dynamically to pathological stimuli and microenvironmental change [13]. Both cell types can accept and degrade compromised mitochondria through transmitophagy and donate functional mitochondria to neurones under stress conditions, including hypoxia, ischaemia, exogenous toxin exposure, and pathological protein aggregation [13–15]. This glia-neuron mitochondrial exchange represents a distinct mechanism for cellular resilience within a suboptimal environment that extends beyond traditional concepts of metabolic coupling.

The clinical relevance of mitochondria transfer has been demonstrated across different neurological conditions. In neurodegenerative diseases, such as Alzheimer’s disease (AD) and Parkinson’s disease (PD), mitochondrial malfunction precedes neuronal death, suggesting a possible therapeutic window for interventions preventing neuronal death [16, 17]. Acute brain injuries, including stroke and traumatic brain injury, involve rapid mitochondrial damage that could potentially be mitigated through enhanced glial-neuronal mitochondria exchange [14, 18]. This review focuses on neuroglia-mediated mitochondria transfer as a disease-modifying mechanism in the nervous system. We propose that neuroglial mitochondria transfer should not be viewed merely as a compensatory rescue response, but as a context-dependent quality-control mechanism. The functional consequences for recipient neurones are shaped by multiple variables, including donor-cell type, donor mitochondrial integrity, recipient-cell metabolic demand or function, and disease progression. Framed in this way, neuroglial mitochondria exchange offers a revised perspective on disease pathogenesis and reveals new avenues for therapeutic intervention in neurological disorders.

## Mitochondria transfer: charting new horizons across cellular borders

### Discovery

**Fig. 1:**
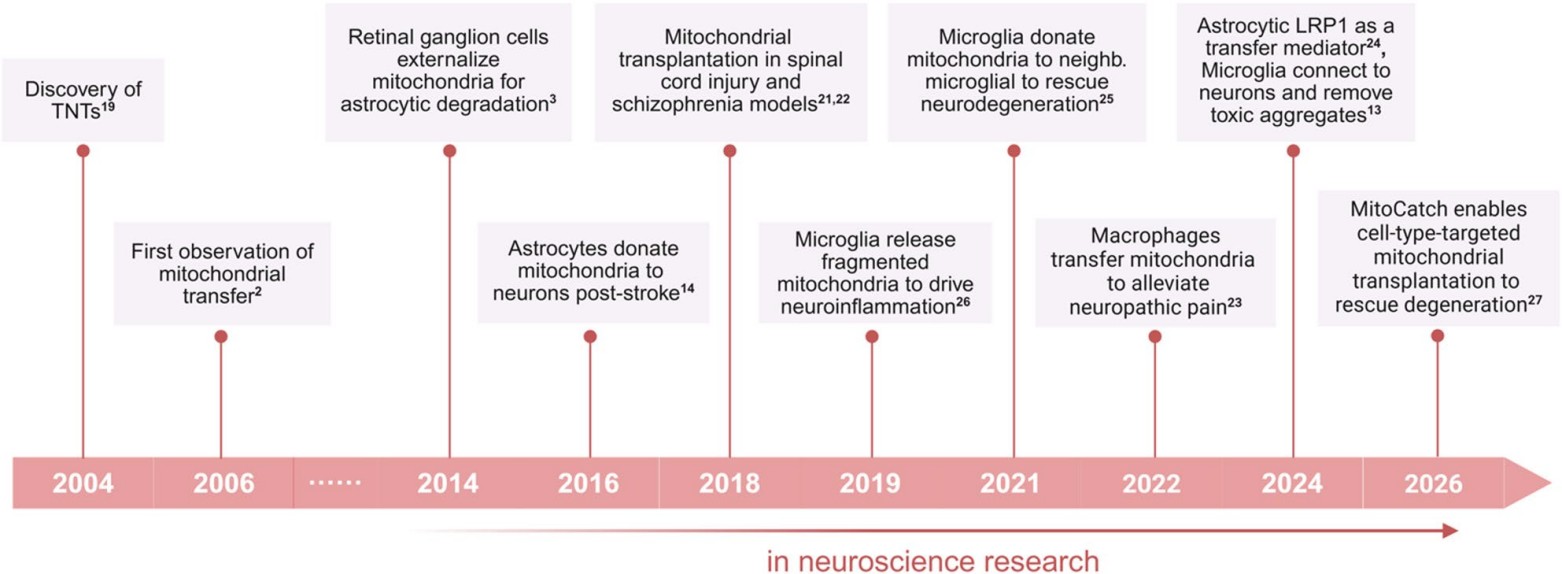
Timeline of discoveries of mitochondria transfer over two decades. This timeline highlights key discoveries in TNTs, mitochondria transfer, and neuroscience, reflecting ongoing research progress in mitochondria transfer

Over the past two decades, studies of mitochondria transfer have advanced our understanding of intercellular communication and adaptation (Fig. 1). The foundation was laid in 2004 with the discovery of tunnelling nanotubes (TNTs), which revealed a novel mechanism for organelle and vesicle exchange between cells [19]. A key breakthrough came in 2006 when functional mitochondria transfer was observed for the first time, demonstrating that whole mitochondria are exchanged between cells to rescue ATP shortage due to aerobic respiration, marking the beginning of the new field [2]. A decade later, astrocytes were found to internalise and degrade mitochondria from retinal ganglion cells after injury, suggesting dysfunctional mitochondria can be transferred from injured neurones to astrocytes for elimination, the process known as transmitophagy [3]. This process however may involve astrocyte phagocytosing damaged cellular components of adjacent neurones [20], suggesting a complex intercellular quality-control mechanism.

The neuroprotective potential of mitochondria transfer was first highlighted by studies demonstrating that astrocytes release extracellular vesicles (EVs) containing functional mitochondria delivered subsequently to ischaemic neurones [14]. This preserved neuronal integrity and demonstrated the therapeutic potential of using exogenous mitochondrial transplantation to protect and rescue neurones during ischaemic injuries [14]. By 2018, several studies had further explored the therapeutic potential of exogenous mitochondria transfer in murine models of spinal cord injury and schizophrenia, reporting benefits such as preservation of mitochondrial respiration and prevention of motor and cognitive deficits [21, 22]. In addition, EV-mediated mitochondria transfer from macrophages to sensory neurones ameliorated neuropathic pain through CD200 receptors (expressed on macrophage-derived EVs) binding to a CD200 receptor ligand iSec1 present in neurones [23]. Furthermore, in response to cerebral artery occlusion, astrocytic low-density lipoprotein receptor-related protein-1 (Lrp1) has been identified as a key mediator facilitating EV-mediated mitochondria transfer from astrocytes to injured neurones critical for functional recovery after ischaemic injury [24]. As the field advanced, both protective and pathological roles of mitochondria transfer have emerged. For example, microglia can donate healthy mitochondria supporting neuronal survival [13, 25], whereas under disease condition, microglia may also deliver fragmented or dysfunctional mitochondria that compromise neuronal function in neurodegenerative diseases [26]. More recently, the development of MitoCatch, a targeted mitochondrial delivery system using cell-type specific protein binders, has overcome a major limitation of non-selective transfer, enabling precise mitochondrial transplantation into retinal ganglion cells, neurons, and photoreceptors to rescue cell degeneration [27]. These findings suggest that intercellular mitochondrial exchange is a tightly regulated, context-dependent process while its targeted manipulation may represent a promising therapeutic strategy for neurological disorders.

### Mechanisms of mitochondria transfer

**Fig. 2:**
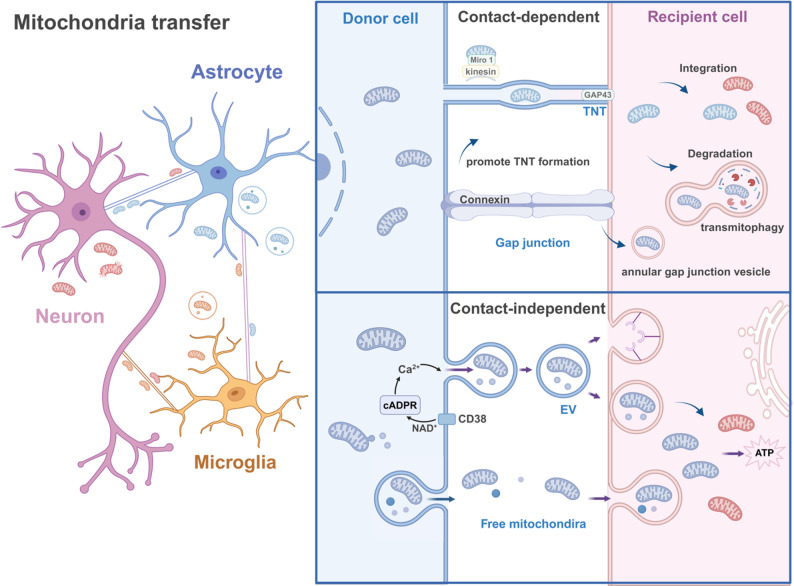
Mechanisms of mitochondria transfer in the nervous system. Donor cells transfer mitochondria through contact-dependent and contact-independent mechanisms. Contact-dependent transfer is mediated by TNTs facilitating direct intercellular mitochondrial exchange. Gap junctions formed by connexin proteins may promote TNTs formation or enable transfer through annular gap junction vesicles. Contact-independent transfer involves EVs that package and transport mitochondria across the cellular membrane. CD38/cADPR/Ca²⁺ pathway promotes fusion of mitochondria-containing vesicles with the donor cell plasma membrane and enhances EV-associated mitochondrial release. Additionally, free mitochondria are directly released into the extracellular space for uptake by recipient cells. Upon capture, transferred mitochondria undergo either functional integration into the mitochondrial network of the recipient cells or quality-controlled degradation by transmitophagy

Mitochondria transfer in the nervous system is a highly regulated and dynamic process, occurring through contact-dependent and contact-independent pathways [28] (Fig. 2). These pathways respond dynamically to the alteration in microenvironment, cellular stress signals, and metabolic demands.

#### Contact-dependent mitochondria transfer

**Fig. 3:**
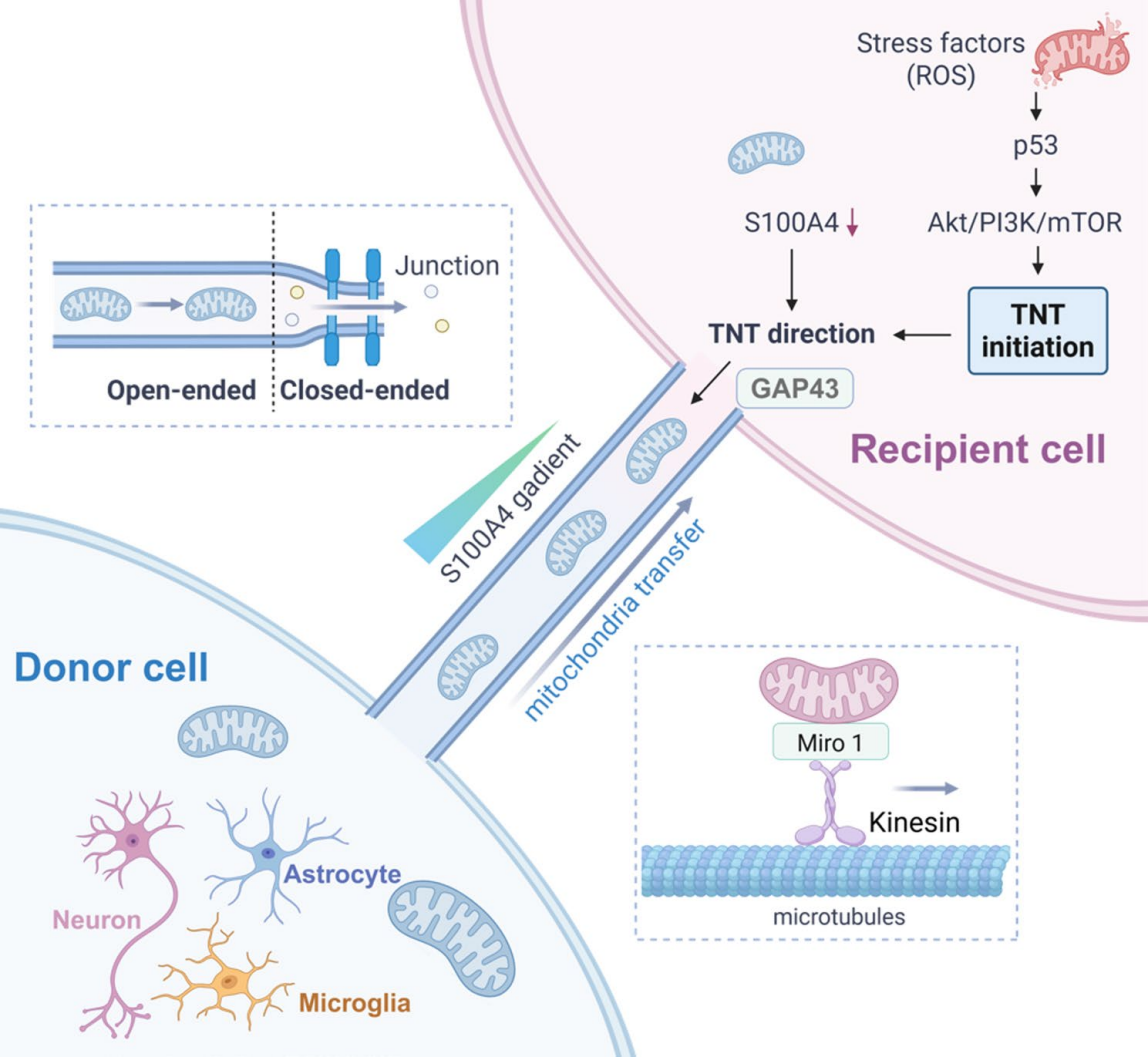
Schematic illustration of TNT structure and molecular regulators. TNTs are classified as open-ended or closed-ended based on membrane continuity. Open-ended TNTs enable direct organelle transfer, including mitochondria, whereas closed-ended TNTs restrict organelle exchange by allowing selective signalling molecules to pass through. Oxidative stress promotes TNT formation through p53-mediated activation of the Akt/PI3K/mTOR pathway, accompanied by actin polymerisation. S100A4 guides TNT extension, while Miro1 facilitates mitochondria transport along TNTs

TNTs, which play a key role in contact-dependent mitochondria transfer, are thin, actin- and microtubule-based cytoplasmic bridges that enable direct intercellular communication over distances ranging from several micrometres to over 100 μm [29, 30] (Fig. 3). In the CNS, TNTs present in neurones and neuroglia function as intercellular ‘transport highways’ and an adaptive mechanism under cellular stress, with their formation markedly increasing under pathological conditions [31]. In models of ischaemic stroke, astrocytes transfer functional mitochondria to energy-deprived or injured neurones by TNTs, thereby promoting neuronal survival [32, 33]. Microglia, on the other hand, employ TNTs not only for mitochondria donation but also for receiving neurotoxic aggregates such as misfolded α-synuclein and hyperphosphorylated tau protein from neurones for degradation, which can reduce neuronal stress and slow disease progression [13, 34].

The formation and regulation of TNTs are closely linked to cytoskeletal dynamics, influenced by Rho family GTPases (e.g., Miro1) and Growth-associated protein 43 (GAP43) [35]. Miro1 regulates mitochondrial trafficking along TNTs by tethering mitochondria to microtubules to ensure their efficient delivery to recipient cells [36, 37]. TNT directional extension is guided by a gradient of the Ca²⁺-binding protein S100A4, with initiating cells exhibiting lower S100A4 levels extending toward target cells with higher S100A4 concentrations [38]. Cellular stressors, such as hypoxia or oxidative damage, further increase TNT formation [32, 39]. However, TNTs have a dual role. They can exacerbate pathological protein, such as α-synuclein, aggregation and accumulation, by transferring α-synuclein to neighbouring cells for aggregation, and propagate the pathology [40–42]. This dual functionality highlights the need for tight regulation of TNT formation to facilitate the transfer of healthy mitochondria while limiting the propagation of toxic protein aggregates.

Adhesion-based gap junctions are primarily formed by connexins such as connexin43 and 30 (Cx43 and Cx30), representing another potential pathway for mitochondrial exchange between adjacent cells [43]. While connexin with ~ 2 nm pore size precludes direct mitochondria passage [44], this structure may facilitate mitochondrial exchange by mediating TNT formation or by assisting the formation of annular gap junction vesicles [45]. Current evidence suggests that Cx43 regulates TNT formation [46]. However, if gap junctions are critical for TNT formation, microglia would theoretically be unable to form TNTs in vivo [47], which contradicts published research observations [25]. Furthermore, if gap junctions can facilitate mitochondria transfer in vivo, dye injection experiments that measure gap junctional communication would detect astrocyte-to-neuron dye transfer, yet such observations have not been reported. Thus, the contribution of gap junctions to neuron-glia mitochondria transfer requires further investigation.

Although TNTs are well characterised in vitro as efficient conduits for mitochondria transfer, their presence and quantitative contribution in vivo remain difficult to define. This limitation largely reflects technical challenges due to tissue architecture and mechanical forces [48] rather than the absence of the phenomenon. TNTs are dynamic and fragile structures (often < 200 nm in diameter) that are easily disrupted by tissue fixation, mechanical sectioning, and physiological brain movements, making them difficult to visualise using conventional histological methods. In vivo imaging approaches such as two-photon microscopy have provided indirect or snapshot-level evidence of TNT-like structures and associated mitochondrial exchange [13, 49], but do not yet allow detailed quantification of transfer frequency or flux. As a result, the functional relevance of TNT in vivo has primarily been inferred from genetic or pharmacological disruption of TNT formation, which consistently attenuates mitochondria transfer-associated neuroprotection in disease models, including stroke and neurodegeneration [13, 50, 51]. Together, these findings support a physiologically meaningful role for TNT-mediated mitochondria transfer in vivo, while highlighting the need for improved real-time, high-resolution imaging, and lineage-tracing strategies to accurately assess its extent under physiological and pathological conditions.

#### Contact-independent mitochondria transfer

EVs are small (30–1000 nm), membrane-bound particles released by cells into the extracellular environment to serve as intercellular communicators by carrying proteins, lipids, nucleic acids, and small organelles [52, 53]. In particular, mitochondria are also packed by EVs to enable intercellular transfer, including intact or damaged mitochondria and mitochondrial DNA (Fig. 2). The EV lipid bilayer membrane protects cargo from degradation during transport between organs at systemic level [52, 54]. In the CNS, astrocytes release EVs containing functional mitochondria, which are internalised by energy-deficient neurones to restore ATP production and support survival [24]. Similarly, in models of multiple sclerosis, neural stem cells were shown to secrete mitochondria-rich EVs that transfer functional mitochondria to mononuclear phagocytes, restoring mitochondrial dynamics and cellular metabolism while reducing pro-inflammatory responses [55]. The activation of CD38/Ca²⁺ pathway increases the loading and release of mitochondria in EVs by converting NAD⁺ into cyclic ADP-ribose (cADPR), which raises cytosolic Ca²⁺ levels and drives vesicle fusion with the plasma membrane [56, 57]. This Ca²⁺-dependent pathway acts as a broad stress-response mechanism; its activation also produces super-donor cells with three-fold higher yield and better mitochondrial quality [56]. Recipient cell uptake of EVs is enhanced during metabolic stress by surface receptor-mediated endocytosis or fusion, although the exact mechanisms require further investigation [58]. Moreover, EVs may carry other bioactive components that protect cells under stress [59]. Taken together, these properties position EVs as promising vehicles for mitochondrial therapeutics, due to their biocompatibility and selective uptake properties [55, 60].

Furthermore, mitochondria release by donor cells and endocytosis by recipient cells represent an alternative mechanism of mitochondria transfer. This process involves direct release of whole or fragmented mitochondria into the extracellular space, with subsequent internalisation by both adjacent and distal recipient cells [61]. This direct release may serve as a rapid adaptive response to neuronal injury, complementing EV-mediated mitochondria transfer during increased oxidative stress and metabolic dysfunction in neurones [62]. However, such uncontrolled direct release of fragmented mitochondria into the extracellular space may exacerbate inflammatory response and oxidative stress, potentially contributing to secondary neuronal injury and degeneration [26]. The balance between adaptive response and pro-inflammatory stimulus represents a critical regulatory checkpoint to determine neurological health.

### Selective mitochondrial sorting for transfer

Under acute injury conditions, donor cells preferentially transfer intact, functional mitochondria to stressed neurones to restore mitochondrial function [50, 63]. This process is regulated by multilayered quality-control mechanisms that selectively package healthy mitochondrial components while excluding damaged or pro-inflammatory cargo. In particular, the OPA1/sorting nexin 9 (Snx9)-dependent pathway facilitates the incorporation of mitochondrial inner membrane and matrix proteins into EVs, while restricting dysfunctional materials and pro-inflammatory signals [64]. In donor cells with impaired mitochondria, Parkin suppresses this OPA1/Snx9 mediated pathway and instead redirects damaged mitochondrial components toward lysosomal degradation rather than EV release, indicating an active selection process that favours the transfer for healthy mitochondria [65].

Miro1 is a rate-limiting spatial regulator. It is highly expressed in competent donor cells, such as mesenchymal stem cells (MSCs), and functions as a Ca²⁺ sensor through its EF-hand domains. Stress-induced cytosolic Ca²⁺ elevation promotes the Miro1-mediated switch from long range microtubule-based trafficking to actin-dependent transport at the TNT entry sites [66], facilitating directed mitochondrial delivery. Mitochondrial membrane potential (ΔΨm) provides an upstream quality control checkpoint. Mitochondria with preserved ΔΨm are preferentially mobilised by Miro1-associated transport complexes, whereas depolarised mitochondria stabilise PINK1, recruit Parkin, and are directed to mitophagy [67]. Together, mitochondria derived vesicle sorting, Miro1-mediated transport gating, ΔΨm surveillance, and stress-responsive signalling converge to ensure the selective transfer of functional mitochondria.

A key unresolved question is why damaged mitochondrial components may still be transferred to recipient cells despite the existence of donor-cell quality-control mechanisms [26]. This may occur when mitochondrial damage exceeds the capacity of mitophagy to recycle and other intracellular clearance pathways to eliminate. In neurodegenerative disease, persistent oxidative stress, impaired mitophagy, and excessive fission may compromise this selective sorting mechanism, allowing damaged mitochondrial fragments, mtDNA, or oxidised mitochondrial proteins to be released or transferred to the neurones and other adjacent cells [26]. This provides a plausible explanation for why mitochondria transfer may contribute to the propagation of inflammatory and degenerative signalling in chronic disease states.

### The fate of transferred mitochondria: integration versus degradation

The quality of transferred mitochondria in recipient cells determines the therapeutic efficacy. Upon uptake, transferred mitochondria face either functional integration into the host mitochondrial network or degradation through mitophagy [68].

During functional integration, free mitochondria or those packaged in EVs are primarily internalised through macropinocytosis, a process dependent on actin cytoskeleton remodeling and PI3K signalling. This process can be pharmacologically blocked by the inhibitor 5-(N-ethyl-N-isopropyl)amiloride (EIPA) that inhibits Na^+^/H^+^ exchangers [69]. Notably, metabolic impairment of recipient cells (e.g., ischaemic or toxin-induced stress) markedly enhances uptake efficiency, thereby facilitating cellular repair through the acquisition of functional mitochondria [70, 71]. As such, the integration of transferred mitochondria is largely influenced by the metabolic state of the recipient cells. Metabolically compromised cells are more likely to retain donor mitochondria to compensate for deficiencies in oxidative phosphorylation and biogenesis capacity. In contrast, metabolically intact cells exhibit comparatively lower endocytotic activity, as exogenous mitochondria are functionally redundant and therefore more likely to be recognised and eliminated through intracellular quality control mechanisms, such as mitophagy [58, 72].

Once integrated, healthy mitochondria enter into the mitochondrial pool of the recipient cells, where they will produce ATP to meet the energy demand to maintain cellular homeostasis [73]. This is supported by the experiment performed in mitochondrial DNA-depleted lung cancer cells (A549 ρ^0^ cells), which are unable to perform oxidative phosphorylation due to abolished mtDNA-encoded subunits of the electron transport chain (complexes I, III, IV, and V). When A549 ρ^0^ cells acquire mitochondria from co-cultured human bone marrow-derived MSCs in vitro, ATP production via oxidative phosphorylation and cell division are restored [2]. Similarly, mitochondria released from neuroglial cells can be transferred to neighbouring neurones to support neuronal energy metabolism, preserve mitochondrial and cellular function, and mitigate cellular injury under ischaemic conditions during a stroke [14, 24]. Mechanistically, these transferred mitochondria may escape endosomal compartments in the recipient cell through mitofusin 1/2 (MFN1/2)-mediated membrane fusion between transferred and endogenous mitochondria, or by directly incorporating into the mitochondrial pool as whole organelles [27, 74].

On the other hand, damaged or metabolically compromised mitochondria are selectively eliminated by transmitophagy, a quality-control process where recipient cells engage their endogenous mitophagy machinery to clear foreign or dysfunctional mitochondrial fragments [75]. Following internalisation, recipient cells recognise compromised mitochondria through characteristic signals such as loss of membrane potential, oxidative damage, or structural abnormalities. These cues activate the PINK1-Parkin pathway, in which PINK1 docks on the outer membrane of impaired mitochondria and recruits the E3 ubiquitin ligase Parkin, leading to ubiquitination of mitochondrial surface proteins [76, 77]. This ubiquitin signalling promotes the recruitment of autophagy adaptors, LC3 lipidation and autophagosome formation, followed by lysosomal degradation upon autophagosome-lysosome fusion [78]. In parallel, the stress-activated mitochondrial metalloprotease OMA1 cleaves OPA1 to suppress mitochondrial fusion and facilitate the isolation of damaged mitochondrial fragments [79], while Parkin-mediated ubiquitination further promotes their segregation and clearance, thereby preventing integration of damaged mitochondria into the healthy mitochondrial network.

In the nervous system, intercellular mitochondria transfer primarily serves as a quality-control and self-maintenance mechanism rather than random organelle sharing. During AD progression, astrocytes can clear dysfunctional neuronal mitochondria received through TNT by activating mitophagy pathways, including upregulation of the autophagy regulator Ambra1 [80]. Similarly, in the PD model, astrocytes degrade damaged mitochondria transferred from dopaminergic neurones, whereby astrocytes complete PINK1/Parkin-mediated clearance of neuronal mitochondria, limiting the propagation of mitochondrial stress [81]. Beyond the brain, similar self-protective strategies are observed in other tissues. For example, brown adipocytes expel oxidatively damaged mitochondria with EVs, which are subsequently taken up and degraded by macrophages to maintain tissue metabolic homeostasis [82]. Related work demonstrates that EV-mediated mitochondrial export represents a form of ‘outsourced mitophagy’ whereby stressed MSCs package partially depolarised mitochondria into LC3-positive microvesicles for macrophage uptake [83]. In this context, macrophages can transiently reuse these mitochondria through fusion, while MSCs simultaneously release microRNA-rich exosomes that dampen macrophage innate immune activation, facilitating safe clearance [83]. Collectively, these findings highlight mitochondria transfer as a regulated homeostatic strategy that balances mitochondrial disposal, reuse, and immune tolerability. Thus, the key translational challenge lies not only in promoting mitochondria transfer, but in controlling cargo quality, recipient-cell processing, and tissue-specific outcomes.

## Mitochondria transfer in diseases

The presence and the potential physiological role of mitochondria transfer in the healthy nervous system remain unclear. It may, for example, assist the biogenesis of new mitochondria in recipient cells, given that several key proteins of the oxidative phosphorylation subunits are coded by mitochondrial DNA translation. Compared with the limited understanding of mitochondria transfer under healthy conditions, the research on neurological disorders is far more advanced, due to the interest in developing novel therapeutics using this mechanism.

### Neurodegenerative diseases

#### AD

AD, a common neurodegenerative disorder, is the leading cause of ageing-related dementia [84, 85]. In human primary cells and AD models, microglia have been found to employ TNTs to facilitate the removal of toxic tau aggregates from neurones and the transfer of healthy mitochondria to these metabolically compromised neurones [13, 31]. This process can inhibit oxidative stress and restore normal neuronal function. These TNTs exist as free-floating structures between neurones and microglia in vitro, while similar formations were observed in mouse and human brains in vivo [13]. Astrocytes also receive and degrade malfunctioning neuronal mitochondria through the TNTs. This process is increased in the brain of aged 5xFAD mice and astrocytes derived from primary iPSCs of patients with familial AD [80]. The neuron-astrocyte mitochondria transfer also prevents the release of pro-apoptotic factors, such as cytochrome c, in neurones to prevent neurodegeneration and maintain energy synthesis function [80].

However, mitochondria exchange between glial cells can promote pathological progression. In disease-associated microenvironment, increased oxidative stress, inflammation, and β-amyloid aggregation can cause severe mitochondrial damage, which induces Drp1/Fis1-mediated fission and subsequent mitophagy. In theory, this process removes damaged mitochondrial fragments, allowing the remaining healthy fragments to fuse and form new functioning mitochondria [86]. However, an excess of dysfunctional mitochondrial fragments can build up in the microglia due to over-fragmentation [87]. These fragments retain outer-membrane markers such as TOM20 and VDAC, but show impaired inner-membrane integrity, cytochrome c release, and are therefore no longer able to maintain normal ATP production. These fragments also act as the damage-associated molecular patterns (DAMPs) and induce sterile innate immune responses in both microglia and neighbouring astrocytes upon uptake [26]. This promotes reactive astrogliosis, characterised by increased pro-inflammatory gene expression and release of TNF-α, IL-1β, IL-6, and IL-1α [26]. Within such a hostile microenvironment, mitochondrial biogenesis is also compromised, in addition to reduced fusion, which further exacerbates energy supply shortage and accelerates neuronal loss. Selective inhibition of the Drp1/Fis1 axis can reduce excessive fission and maintain a balanced mitochondrial quality control [26]. In addition to dysfunctional mitochondrial fragments, extracellular or circulating cell-free mitochondrial DNA (ccf-mtDNA) from stressed glial cells and neurones may also act as DAMPs and activate innate immune pathways in microglia and astrocytes [88, 89]. However, the relative contribution of ccf-mtDNA versus transferred mitochondrial fragments to neural inflammation in AD remains to be fully defined.

On the other hand, the transplantation of exogenous healthy mitochondria shows promise for AD treatment. Intravenous infusion of fresh active mitochondria isolated from HeLa cells into AD mice improved cognitive function, reduced neuronal loss and gliosis in the hippocampus [90]. Furthermore, EVs derived from MSCs that carry functional mitochondria alleviated oxidative stress and restored mitochondrial function in SH-SY5Y neuroblastoma cells treated with okadaic acid, which is an in vitro model of AD mimicking tau hyperphosphorylation [52]. However, as MSC EVs also contain other cargo, such as miRNA and proteins, it is difficult to determine whether observed beneficial effects were solely due to functional mitochondria. Nevertheless, these preclinical studies suggest that functional mitochondria alone or carried with stem cell EVs are plausible new options for future clinical trials in rescuing neurones in patients with AD.

#### PD

PD is the second most prevalent neurodegenerative disorder, characterised by the progressive loss of dopaminergic neurones in the substantia nigra and the accumulation of pathological α-synuclein proteins that form Lewy bodies [91]. These hallmark features are accompanied by the heightened vulnerability of dopaminergic neurones to oxidative stress and increased metabolic demands [91]. Mitochondrial malfunction plays a central role in PD pathogenesis, and therefore, intercellular mitochondria transfer has emerged as a potential strategy for mitigating neuronal damage at early stages of the disease [92, 93].

In PD, microglia transfer healthy mitochondria to stressed neurones, particularly in response to α-synuclein accumulation [13, 34]. α-Synuclein aggregates within stressed neurones induce TNT formation, which facilitates the transfer of α-synuclein to microglia for degradation [13, 34]. In return, microglia donate their functional mitochondria to the stressed neurones [13, 34]. The Rac-PAK signalling pathway, regulated by the P2Y_12_ receptor, has been reported to mediate TNT formation between neurones and microglia in PD [13]. Similarly, delivery of astrocyte-derived mitochondria to affected neurones can attenuate rotenone-induced dopaminergic neurodegeneration and axonal damage in vitro [94]. Unlike the canonical TNT-mediated pathway, this process is regulated by the p38 MAPK signalling cascade, inhibition of which disrupts mitochondrial internalisation by neurones in an in vitro model of PD [94].

Mitochondria transfer between neuroglial cells may also contribute to the alleviation of PD pathology. In the acute cell model of PD, F-actin-dependent TNTs facilitate α-synuclein fibrils transfer from overloaded microglia to less burdened neighbouring microglia, which in return donate functional intact mitochondria to overloaded microglia to attenuate inflammation-induced cytotoxicity [25]. PD-associated genetic mutations, such as TREM2 or LRRK2, can impair TNT formation and mitochondrial dynamics, compromising microglial protective effects [25]. Similarly, stressed astrocytes transfer α-synuclein aggregates to healthy ones through TNTs in exchange for functional mitochondria to restore mitochondrial function in stressed astrocytes [95]. These bidirectional transfers alleviate cell stress and support normal glial cell function, highlighting the cooperation in neuroglial networks. However, these adaptations may only function at the early stage of PD; once PD pathology has been fully developed, mitochondrial function is compromised in all cell types in the CNS, and thus, the exchange of aberrant mitochondria may accelerate PD progression. Furthermore, transferring platelet-derived mitochondria from PD patients into mitochondrial DNA-depleted NT2 cells can induce PD-like abnormalities, including reduced mitochondrial complex I activity, reduced ATP production, metabolic dysregulation, and increased NF-κB signalling, suggestive of inflammatory responses [96, 97]. Together, these findings indicate that neuroglial mitochondria transfer exerts stage-dependent effects in PD, acting as a protective, stress-buffering mechanism early in disease but becoming maladaptive as mitochondrial malfunction becomes widespread. In this latter context, the exchange of damaged mitochondria and pathological cargo may amplify inflammatory signalling and metabolic failure, thereby accelerating progression of neurodegeneration.

Recent studies clarified how PD-associated mutations impair protective mitochondria transfer. In iPSC-derived models carrying the LRRK2 G2019S variant, mitochondrial release was reduced in astrocytes in response to the oxidative stress induced by rotenone [98]. This is due to the hyperactive LRRK2 kinase that increases Drp1 phosphorylation at Ser616, which promotes detachment of STX17, a mitochondrial outer-membrane protein involved in mitochondrial export [98]. As a result, mitochondria transfer from astrocytes to dopaminergic neurones is reduced, thereby facilitating neuronal injury [98]. This finding reveals a cell-type-specific vulnerability in PD: astrocytes, normally serve as protective mitochondrial donors, exhibit impaired mitochondria transfer capacity under pathological stress. Whether this impairment is also accompanied by the transfer of aberrant mitochondria remains to be determined. Therefore, strategies that enhance mitochondria transfer without addressing donor-cell dysfunction or mitochondrial quality may be ineffective or even detrimental.

#### Multiple sclerosis (MS)

In MS, mitochondrial density is markedly reduced in demyelinated axons, prompting neurones to transport functional mitochondria from the soma to energy-starved axons as an adaptive response to demyelination [99]. This endogenous adaptive mechanism, however, is often insufficient, necessitating exogenous mitochondrial transplantation [99]. Emerging evidence indicates that cultured neural stem cells provide neuroprotection through EV-mediated delivery of functional mitochondria [55]. Proteomic and structural analyses also confirm the enrichment of metabolically active mitochondria within neural stem cell-derived EVs [55]. The incorporation of these mitochondria into inflammatory macrophages can restore cellular metabolism and reduce pro-inflammatory markers in vitro [55]. In the experimental autoimmune encephalomyelitis model of MS, intracerebroventricular administration of either neural stem cells or their EVs resulted in mitochondria transfer to macrophages and astrocytes. This was associated with improved behavioural outcomes [55]. However, given that EVs carry a diverse repertoire of bioactive cargo, including proteins, lipids, and nucleic acids, additional EV-associated components are likely to contribute to the observed therapeutic effects, and their relative roles warrant further investigation.

### Stroke

Ischaemic stroke is caused by cerebral artery occlusion resulting in hypoxia and ischaemia, and triggering a cascade of pathological events, including mitochondrial damage [100]. Mitochondria transfer from endogenous neuroglia and exogenous stem cells has emerged as a key mechanism to rescue neuronal function and promote recovery [101, 102]. In ischaemic environments, astrocytes exhibit remarkable adaptability and are recognised as active donors of mitochondria transfer, providing essential energy support to injured neurones [14, 24, 103]. Transient focal cerebral ischaemia in mice prompted astrocytic mitochondrial relocation into adjacent neurones [14]. This astrocyte-neurone transfer operates through two molecular pathways: CD38 and Lrp1 signalling. In astrocytes, CD38-cADPR-driven Ca²⁺ signalling promotes Ca^2+^-dependent release of functional mitochondria. On the other hand, CD38 also stimulates Endoplasmic Reticulum (ER)-mitochondria interactions through ryanodine receptors, facilitating mitochondrial Ca^2+^ uptake and promoting the secretory capacity of mitochondria [103]. Furthermore, in a mouse model of ischaemia-reperfusion injury, astrocytic Lrp1, an endocytic signalling cell-surface receptor, suppressed glycolysis and lactate production, which reduces lactylation of ADP-ribosylation factor 1 (ARF1), a cytosolic GTPase involved in vesicle trafficking [24]. This enhances the transfer of functional mitochondria from astrocytes to neurones, thereby ameliorating ischaemic stroke injury [24]. Knockdown of Lrp1 reduced astrocyte-derived mitochondria transfer to neurones in vivo, an effect that can be mitigated through direct intracerebroventricular mitochondrial infusion [24].

Building upon these discoveries, several therapeutic interventions have been developed aiming to enhance astrocyte-mediated mitochondria transfer. Pharmacological approaches include chrysophanol, ginsenoside Rb1, and JZL-184, which regulate fatty acid metabolism by promoting TGF-β1-dependent mitochondria transfer [104–106]. Mild hypothermia therapy also showed promise by enhancing mitochondria transfer from astrocytes to damaged neurones through increased TNT formation [33] and enhancing functional mitochondria release into the extracellular space to facilitate indirect transfer [107]. Ca^2+^-binding protein S100A9 increased the uptake of transplanted mitochondria in microglia, which promotes redox homeostasis and anti-inflammatory effects [108]. Microvesicles and EVs are efficient vehicles for mitochondria transfer to brain endothelial cells, which can restore ATP production and BBB integrity under ischaemic conditions [109]. Notably, medium-to-large size EVs exhibit greater efficacy than small EVs in reducing infarct volume and enhancing BBB protection, particularly as binary mixtures with heat shock protein 27 (HSP27), which has been shown to preserve tight-junction integrity of brain endothelial cells [110].

Neural stem cells and MSCs are also key mitochondrial donors for exogenous mitochondria transplantation [111–113]. Neural stem cells derived from foetal human brain transferred functional mitochondria to ischaemic neuronal cells in vitro, such as differentiated SH-SY5Y, via TNTs, and reduced oxidative stress-induced apoptosis [114]. In a mouse model of middle cerebral artery occlusion and a cell model of oxygen-glucose deprivation / reoxygenation, functional mitochondria isolated from MSCs were preferentially internalised by neurones and endothelial cells thus reducing reactive oxygen species (ROS) levels and improving cell survival [115]. Overexpression of Miro1 in MSCs can increase mitochondria transfer through TNT and improve neurological outcomes in stroke models [116, 117]. Furthermore, sirtuin1 (SIRT-1), an NAD^+^-dependent deacetylase, deacetylated Mitofusin 2 protein (MFN-2) to facilitate TNT formation and related mitochondria transfer [118]. SIRT-1 itself can enhance mitochondrial fusion and biogenesis, potentially supporting long-term cellular metabolic function [118].

In haemorrhage stroke, such as intracerebral haemorrhage (ICH) [119], astrocytes secrete functional mitochondria into the extracellular space, which, when internalised by microglia, can switch the latter towards a reparative phenotype [120]. This improved hematoma absorption and promoted the repair of injured brain tissue after ICH [120]. Additionally, intravenous transplantation of astrocytic mitochondria can improve neuronal antioxidant defences and neuroplasticity, supporting neurological functional recovery [121]. A key mediator in these processes is humanin, a mitochondria-derived peptide that activates signal transducer and activator of transcription 3 (STAT3) to upregulate mitochondrial endogenous antioxidant manganese superoxide dismutase to boost their antioxidant capacity [120, 121]. In mice with ICH, humanin reduced neurological deficits and accelerated hematoma absorption, highlighting its therapeutic potential [120].

Circulating extracellular mitochondria and ccf-mtDNA have been recognised as biomarkers of neurological injury [122]. In subarachnoid haemorrhage stroke, higher mitochondrial membrane potential in the cerebrospinal fluid (assessed directly by flow cytometry) correlates with improved neurological outcomes, which may indirectly reflect improved repair of injured neurones by mitochondria [123]. Similarly, EAAT1-positive mitochondria (presumably astrocyte-derived) in cerebrospinal fluid may serve as a prognostic biomarker indicating the outcome of subarachnoid haemorrhage [123]. Based on these findings, increasing endogenous mitochondria transfer from neuroglia to neurones or supplementation of exogenous mitochondria may offer a novel approach to mitigate oxidative stress and inflammation and promote the restoration of neurological function.

### Brain and spinal cord injury

Traumatic brain injury (TBI) and surgical brain injury remain the leading causes of disability worldwide [124]. During the acute phase of TBI, there is reactive proliferation and hypertrophy of astrocytes in response to ischaemia and hypoxia [125]. Reactive astrocytes increase mitochondria transfer to damaged neurones, facilitated by increased Cx43 expression and TNT formation to facilitate the mitochondria transfer [126]. Additionally, exogenous mitochondria transplantation provides effective treatment strategy [127]. Transplantation of mitochondria derived from allogeneic liver or autogeneic muscle in the injured cortex reduced neuronal apoptosis and increased the level of brain-derived neurotrophic factor in reactive astrocytes, which promotes the recovery of neurocognitive functions, particularly spatial memory and anxiety-related behaviours [127]. In surgical brain injury, the implantation of hydrogel with MSCs at injury sites led to mitochondria transfer from MSCs to injured neurones ameliorating neuronal mitochondrial injury and resulting in improvement in oxidative stress and neuroinflammation [128]. However, clinical translation of this approach faces the challenge of open-skull surgeries.

Spinal cord injury causes severe, lifelong disability through an initial mechanical trauma, followed by motor, sensory, and autonomic impairment [129]. Mitochondrial malfunction exacerbates neuronal death, axonal degeneration, and myelin loss. Zinc, known for its antioxidant and neuroprotective properties, facilitated mitochondria transfer from microglia to neurones through the SIRT3/Mfn2-dependent pathway [130]. This transfer resulted in increased mitochondrial density and ATP production in neurones, with reduced oxidative stress and improved functional recovery post-spinal cord injury [130]. It also needs to be noted that zinc has multiple effects, where it can directly benefit mitochondrial function by reducing oxidative stress.

Recently, MSC-mediated mitochondria transfer has gained attention as a therapeutic approach. It is driven by CD157-regulated cADPR-Ca^2+^ signalling pathway that can promote bone marrow-derived MSCs (BMSCs) derived mitochondria transfer to injured ventral spinal cord 4.1 (VSC4.1) motor neurones in vitro, resulting in reduced neuronal apoptosis [131]. Transplantation of CD157-modified BMSCs at the injured sites can also improve axonal regeneration and functional recovery in rats with spinal cord injury [131]. Strategies, such as photobiomodulation, enhances Cx36-dependent mitochondria transfer to neurones in spinal cord injury models, leading to improved functional outcomes [132]. Furthermore, Prussian blue nanozyme-anchored MSCs (HQ-Mitofactories) allow continuous production and targeted delivery of functional mitochondria to injured neurones in the spinal cord, to restore mitochondrial homeostasis in recipient neurones and stem cells, therefore enhancing neuronal regeneration and remyelination, associated with improved motor function [133]. Collectively, these studies suggest the therapeutic potential of MSC-based strategies to restore mitochondrial function after spinal cord injury, while also highlighting the importance of precisely regulating mitochondrial delivery, quality, and targeting to maximise functional repair.

### Epilepsy

Epilepsy is a chronic neurological disorder affecting approximately 1% of the global population. It remains a major challenge in both neuroscience research and clinical management due to its complex pathophysiology and significant morbidity [125]. The relationship between mitochondrial damage and epilepsy is bidirectional. Epilepsy induces oxidative stress through excessive neuronal excitation which increases ROS production that in turn impairs mitochondria, while mitochondrial dysfunction exacerbates neuronal hyperexcitability, creating a vicious cycle [134]. HSDL2 is a lipid-metabolic regulator in astrocytes that can protect mitochondrial integrity. It is increased in temporal lobe epilepsy as an adaptive response, highlighting the link between mitochondrial malfunction and epileptogenesis [135]. Moreover, HSDL2 may serve as a potential biomarker for diagnosing temporal lobe epilepsy [135].

A dysregulation in the mitophagy can cause abnormal neuronal discharge and epileptic seizures [136]. Overactivated mitophagy can lead to extensive mitochondrial removal, thus reducing ATP production and resulting in the destabilisation of neuronal membrane potential with an increased risk of abnormal neuronal discharge [137]. Glutaminase 2 (GLS2) reduced seizure activity by inhibiting excessive mitophagy through interaction with mitophagy-related proteins, including PINK1 and LC3, thereby preserving mitochondrial populations and restoring energy metabolism [136]. This highlights the importance of tightly regulated mitochondrial quality control for maintaining normal neuronal function [136]. Furthermore, damage to astrocyte mitochondria can impair ATP metabolism, glutamate homeostasis, and Ca^2+^ signalling, which contribute to the onset of epilepsy symptoms [138]. Given the limited direct evidence of astrocyte-to-neurone mitochondria transfer in epilepsy, further studies are required to determine whether this process occurs during seizures and to reveal its underlying mechanisms.

### Peripheral neuropathy

Mitochondria transfer can benefit peripheral neuropathy, inhibiting inflammatory pain and nerve injury. During inflammatory pain, macrophages transfer mitochondria to dorsal root ganglion sensory neurones by EVs, which restores oxidative phosphorylation and Ca^2+^ homeostasis and suppresses inflammation, thereby alleviating pain [23, 139]. The resolution of pain by mitochondria transfer is mediated by the recognition and binding between the CD200 receptor (CD200R) on macrophages and the CD200R-ligand iSec1 on sensory neurones. Notably, CD200R absence in macrophages or inhibiting iSec1 on neurones both diminished analgesia, suggesting a novel target for the potential development of treatment strategies [23]. In a mouse model of chemotherapy (oxaliplatin)-induced peripheral neuropathy, transplantation of exogenous mitochondria prevented the development of mechanical and cold allodynia by suppressing ERK1/2 activation induced neuroinflammation in the spinal cord [140]. This approach also reduced oxaliplatin-induced weight loss, demonstrating a possible adjuvant therapy with chemotherapy to maintain overall health [140]. Furthermore, under physiological conditions, satellite glial cells (SGCs [141]) transfer mitochondria to dorsal root ganglion sensory neurones through SGC-derived, myosin 10-dependent TNTs [51]. This transfer is impaired in both diabetic and chemotherapy-induced peripheral neuropathy [51]. As a result, restoring SGC-neuron mitochondria transfer protects against nerve degeneration and alleviates neuropathic pain in chemotherapy-induced and diabetic models [51]. However, key questions remain unresolved. Why do SGCs suppress mitochondria transfer? Does this suppression reflect a prioritisation of SGC self-maintenance over neuronal support under energy crisis conditions, or does impaired mitochondria transfer drive the development of neuropathy rather than a result of disease progression?

Following sciatic nerve injury, TNTs form between Schwann cells and axons, facilitating intercellular communication and transfer of neurotropic factors, proteins, mitochondria, and RNA [142]. This process is regulated by small GTPases Rab8a and Rab11a, the knockdown of which impairs TNTs formation and vesicle transfer, resulting in compromised Schwann cell survival and axonal outgrowth from dorsal root ganglion neurones [142]. In mice with sciatic nerve injury, the administration of mitochondria into the injured sciatic nerves promoted axonal regeneration through retrograde transport to dorsal root ganglia, where they upregulate Atf3 and other regeneration-associated genes, such as Sox11 and Smad1 [143]. In retinal degenerative diseases, cone photoreceptors maintain mitochondrial homeostasis by transferring damaged mitochondria to Müller glial cells, to preserve visual functions [144]. However, this serves as a homeostatic buffer rather than an absolute defence: it mitigates acute stress but cannot sustain protection when chronic damage exceeds Müller glial clearance capacity.

### Psychiatric disorders

Mitochondrial malfunction, including reduced ATP synthesis, mtDNA abnormalities and impaired Ca^2+^ regulation, is documented in psychiatric disorders; it affects neuronal energy metabolism, synaptic plasticity, and stress responses [145]. Therefore, mitochondrial transplantation was tested as a treatment strategy for some psychiatric disorders. For example, in models of schizophrenia, functional mitochondria transplant improved the differentiation of patient-derived iPSCs into glutamatergic neurones, demonstrated by increased neuronal markers (β3-tubulin, synapsin1, and Tbr1) and improved glutamate-glutamine cycle activity [146]. In rats, injection of mitochondria into the prefrontal cortex maintains mitochondrial membrane potential in neurones and reduces attention deficits associated with schizophrenia, indicating potential cognitive benefits [146]. While the research is limited, further studies are required to clarify the underlying mechanisms and to determine the broader physiological significance of mitochondria transfer in other psychiatric disorders.

### Ageing

Mitochondrial malfunction is a key player in ageing and age-associated diseases, including neurodegeneration [16, 147]. Mitochondria transfer may counteract ageing-associated cellular decline by reducing senescence phenotypes, restoring proliferation, and improving oxidative phosphorylation and ATP production [148]. For instance, Dmp1-expressing astrocytes transfer mitochondria to endothelial cells via their endfeet for maintaining BBB integrity in Mitofusin 2 (MFN2)-dependent manner [149]. In middle age (7-month-old), astrocytes enhance mitochondria transfer to brain endothelial cells and pericytes at the BBB, potentially as a compensatory response to perceived mitochondrial stress in endothelial cells during ageing [73]. Nevertheless, in advanced ageing (20-month-old), astrocytic MFN2 expression declines subsequently reducing mitochondria transfer efficiency despite a persistent decrease in healthy mitochondria in stressed endothelial cells [149]. This creates a mismatch between the recipient cells demand and reduced donor capacity, which may contribute to BBB damage [149].

The quality of transferred mitochondria is compromised during ageing. During senescence, which is a normal process of ageing, dysfunctional mitochondria can accumulate within senescent cells, including astrocytes and neurones. mTOR/CDC42, which is dysregulated in cellular senescence, drives TNTs formation to facilitate aberrant mitochondria transfer from senescent cells, potentially spreading oxidative stress to the recipient cells [150, 151]. In contrast, exogenous MSC-derived EVs, administered by tail vein injection in D-gal-induced ageing mice, can deliver functional mitochondria to senescent cells, thus restoring mitochondrial biogenesis, reducing oxidative stress, and suppressing senescence-associated secretory phenotype-related inflammation, as demonstrated by decreased IL-1β, P21 expression, reduced SA-β-gal activity, and improved antioxidant enzyme levels [152]. Observed benefits may not be attributed solely to functional donor mitochondria. Additional cargo within the EVs can also contribute to these effects. In addition, whether transplanting exogenous mitochondria, such as those from healthy donors or as part of stem cell derived EVs, can serve long-term benefits in chronic ageing requires further validation [153].

### Methodological challenges

Recent advances in mitochondria transfer research have provided new insights into its biological significance. However, substantial technical challenges remain, particularly in the real-time in vivo analysis of mitochondria transfer dynamics.

 In vitro co-culture studies show that respiration-deficient ρ^0^ cells can regain the ability of aerobic glucose metabolism through acquiring healthy mitochondria [2]. However, these results are difficult to be confirmed in vivo due to complex interactions between multiple cell types [154]. Although models like organoid systems and 3D cultures were developed to include multiple cell types and spatial structure, their authentication is still not comparable to the in vivo environment, as organoids lack vascular perfusion and immune surveillance. Fluorescence-based tracking methods, although widely used, can confound genuine mitochondria transfer events with experimental artifacts [155]. Improved techniques, including photoactivatable markers, reporter proteins, and advanced imaging (e.g. confocal microscopy and intravital two-photon imaging), offer greater specificity for detecting mitochondria transfer events but still face challenges in reliably quantifying transfer efficiency [24]. At the molecular level, analysis of mitochondrial DNA in recipient cells can provide evidence of transferred mitochondria; however, confirming mitochondrial integrity and functional integration remains difficult [156]. Functional assays, including measurement of oxygen consumption and ATP production, can partially address this limitation by assessing the metabolic consequences of mitochondria transfer.

Flow cytometry enables high-throughput quantification of extracellular mitochondria [14], although this approach yields limited mechanistic information on transfer routes and molecular regulators. Examining TNT-mediated transfer in brain tissue and studying EVs as mitochondrial carriers represent promising avenues for advancing the field. Transmission electron microscopy identified TNT-like, mitochondria-containing structures in both mouse and human dorsal root ganglia, extending more than 30 μm deep into tissue [51]. However, definitive stratification of these structures remains constrained by the lack of immune-labelling for F-actin or specific membrane markers, as well as the technical challenges associated with real-time imaging in living tissue.

EVs were validated as carriers of mitochondria through complementary morphological and functional approaches. Cryogenic transmission electron microscopy enables direct visualisation of mitochondria within EVs, revealing characteristic double membranes and cristae, while nanoflow cytometry enables high-throughput analysis of EV size and mitochondrial cargo [157]. Functional assays further demonstrated that EVs-resident mitochondria retain oxidative phosphorylation capacity, as evidenced by preserved ATP synthase activity and functional respiratory chain [55]. Consistent with this, rescue experiments show that stem cell-derived EVs can deliver functional mitochondria to recipient cells, reversing respiratory defects and reducing oxidative stress [14]. However, technical challenges for EV studies remain, including definitive authentication of intact mitochondria-containing EVs from mitochondrial fragments or contaminants, quantifying mitochondrial loading efficiency, and tracking the fate and functional integration of EV-delivered mitochondria in vivo.

To reduce overinterpretation, future studies may benefit from more clearly defined criteria for evaluating functional mitochondria transfer. First, transferred material should be characterised by high-resolution imaging to verify whether it represents intact mitochondria or functional mitochondrial fragments, with donor origin established through genetic or photoactivatable labelling rather than nonspecific dyes. Second, recipient cell mitochondrial uptake should be distinguished from surface adhesion or debris phagocytosis. Most importantly, functional relevance should be supported by assay that directly report mitochondrial activity, such as respiration assays, membrane-potential measurements, or loss of transfer controls, rather than inferred solely from downstream phenotypic changes. Adoption of such approaches will improve reproducibility and help distinguish bona fide mitochondria transfer from related but mechanistically distinct processes.

## Therapeutic potential of mitochondria transfer and future perspectives

### Therapeutic strategies

Recent research demonstrates that certain drugs and small molecules can promote mitochondria transfer from glial cells or exogenous MSCs to neurones. This mechanism of action may partially contribute to their therapeutic effects to improve neuronal survival and limit tissue damage, especially in acute neurological conditions, such as stroke. For example, Chrysophanol and Ginsenoside Rb1 boost astrocyte-to-neurone mitochondria transfer, supporting neurological recovery after ischaemic injury [104, 105]. Zinc aids microglia-to-neurone mitochondria transfer through the SIRT3/Mfn2 pathway during spinal cord repair [130], while melatonin enhances this process through increased formation of TNTs which reduces oxidative stress [158, 159]. In addition, agents such as zinc and melatonin can exert a broad range of beneficial effects on the microenvironment, including anti-inflammatory, anti-apoptotic, and regulating metabolism, thus supporting tissue repair through mechanisms that extend beyond simply restoring mitochondrial function, which is also key to meet the energy demand during this process.

Neural stem cells, MSCs, and hematopoietic stem cells are effective mitochondrial donors. Transplanted neural stem cells can transfer functional mitochondria by EVs to improve energy metabolism and reduce neuroinflammation in recipient cells [55]. Similarly, exogenous MSC transplant can deliver healthy mitochondria to injured neurones through TNTs to reduce oxidative stress and apoptosis [118]. The SIRT-1/RHOT-1/PGC-1α pathway regulates mitochondrial biogenesis and TNT formation, activation of which can improve neuronal energy metabolism and provide neuroprotection [118]. Furthermore, in rat models of spinal cord injury, bone marrow-derived MSCs injected into the epicentre of injury transferred functional mitochondria to motor neurones thus promoting neuronal survival and improving motor function [160]. Transplantation of hematopoietic stem cells also mitigated mitochondrial dysfunction in genetic disorders such as Friedreich’s ataxia [161]. Recent advances in cellular engineering improved mitochondrial stability, targeted delivery, and functionality through genetic modification, biomaterial scaffolds, and microenvironmental modulation. For example, in a model of spinal cord injury, upregulating CD157 in MSCs enhances mitochondrial biogenesis and transfer which improved axonal regeneration [131]. Hydrogels can provide a 3D spatial structure to facilitate mitochondria transfer from transplanted MSCs to injured neurones, and reduce inflammation and oxidative stress in traumatic brain injury [128].

Direct transplantation of healthy, functional mitochondria was considered as a potential therapeutic strategy. This approach requires the isolation, optimisation, and administration of viable, functional mitochondria [162]. In preclinical models of AD and PD, mitochondria derived from human MSCs demonstrated both neuroprotective and anti-inflammatory properties, with improvements in dopaminergic neuronal function and behavioural outcomes [163]. Intravenous delivery of freshly isolated human mitochondria in AD models enhanced cognitive performance and reduced neuronal loss [90]. In cerebral ischaemia, both intravenous and local injection of exogenous mitochondria resulted in integration of the latter with host cells, reduction in infarct size, suppression of inflammation, and stimulation of neurogenesis [108, 164]. In models of traumatic brain injury and surgical brain injury, mitochondria from muscles supported ATP synthesis and contributed to neuronal recovery [21]. The efficacy of mitochondrial transplantation depends on the isolation and delivery protocols [165]. High-quality mitochondria are generally obtained from autologous tissues, such as skeletal muscle, liver, or stem cells, through mechanical disruption and gradient centrifugation [166], however, these conventional isolation procedures may compromise the integrity and function of mitochondria. Thus, the development of advanced techniques is needed, including mitochondrial preconditioning and surface modification, in order to enhance therapeutic efficacy. Surface modification can improve transplantation outcomes by enhancing stability, biocompatibility, and targeted delivery. Peptide-mediated modifications, such as cell-penetrating peptides, help mitochondria enter recipient cells to restore respiration and ATP production [167]. Advanced techniques, such as nanoenzyme-anchored mitofactories, have been shown to scavenge ROS, enhance transplantation efficiency and improve neuronal recovery in spinal cord injury [133]. Polymer coatings, such as dextran-triphenylphosphonium conjugates, may boost mitochondrial stability and uptake [168], while artificial lipid membranes mimic natural bilipid cell membranes to improve biocompatibility and targeted delivery of mitochondria, while reducing degradation [169]. EVs are effective carriers for functional mitochondria. Neural stem cell-derived EVs support mitochondria transfer and restore mitochondrial membrane potential and respiratory function in recipient cells [55]. Homotypic EVs usually have a high transfer efficiency [170]. However, scalability is a key limitation for using EV from primary cells. In this regard, engineered or synthetic EVs may offer better and scalable delivery options [171]. These findings suggest mitochondria transfer as a promising therapeutic target for neurological diseases. Collectively, these advances highlight mitochondria transfer as an innovative and promising therapeutic strategy with the potential to transform the management and outcomes of neurological disorders.

### Clinical applications

#### Clinical trials

Several mitochondrial transplantation trials are currently registered on ClinicalTrials.gov and are summarised in Table 1. In neurological disorders, NCT04998357, led by Washington University, represents the first trial (posted on ClinicalTrials.gov on August 10, 2021) investigating the safety of mitochondrial delivery for acute ischaemic stroke [172]. In this study, autologous mitochondria isolated from skeletal muscle at the vascular access site during standard endovascular reperfusion, are infused intra-arterially through a microcatheter directly into the ischaemic area. Phase 1 results were published in 2026, reporting interim data from 4 participants (3 females, 1 male; aged 43–80 years). The primary focus was safety and feasibility of intra-arterial autologous mitochondrial delivery during mechanical thrombectomy. No adverse events were noted, and the isolated mitochondria maintained viable morphology and functional integrity. Although this trial only examined the safety of mitochondria transplantation, it marks an important initial step toward clinical translation of mitochondrial-based therapies in neurological disease.

In cardiac disease, the first phase 1 clinical trial was reported in 2017 [173]. Five paediatric patients with ischaemia–reperfusion injury requiring extracorporeal membrane oxygenation (ECMO) received epicardial injection of autologous mitochondria isolated from healthy skeletal muscle. Four of five patients showed improved ventricular function and were successfully withdrawn from ECMO, with no reported transplantation-related arrhythmia, haematoma, or scarring [173]. Other registered studies include direct injection of autologous mitochondria into the ischaemic myocardium in paediatric patients on ECMO support (NCT02851758), intravenous allogeneic umbilical cord MSC-derived mitochondria for refractory polymyositis/dermatomyositis (NCT04976140, Phase I/IIa), and autologous haematopoietic stem cell-enriched mitochondria for Pearson marrow–pancreas syndrome (NCT03384420, completed). A combined approach using autologous skeletal muscle mitochondria and MSC-derived exosomes is also being tested in patients undergoing coronary artery bypass grafting with left ventricular dysfunction (NCT05669144, Phase I/II). Collectively, these studies show that mitochondrial transplantation therapies have entered early clinical investigation. However, evidence is still limited to small-scale, early-phase, mostly non-randomised trials, so conclusions on efficacy remain premature.

**Table 1:** Summary of mitochondrial transplantation clinical trials

Disease/ Indication	Mitochon-dria Source	Delivery Route and dose	Phase	Status/ Locations	NCT Number	Key Findings/Notes
Acute ischaemic stroke	Autologous skeletal muscle	Intra-arterial microcatheter infusion (during reperfusion)/ 7 × 10⁶ mitochondria	N/A (2021–2026)	Recruiting/ Seattle, Washington, United States	NCT04998357 [172]	First-in-human brain trial, safety, and feasibility of autologous mitochondrial transplantation
Ischaemia-reperfusion injury requiring extracorporeal membrane oxygenation	Autologous skeletal muscle (rectus abdominis)	Epicardial injection/ 1 × 10⁸ ± 1 × 10⁵ mitochondria	I (2017)	Completed/ Boston, Massachusetts, United States	Emani et al., 2017 [173]	First clinical application, 4/5 patients showed improved ventricular function and withdrew from ECMO; no reported transplantation-related arrhythmia, haematoma, or scarring
Ischaemia-reperfusion injury requiring extracorporeal membrane oxygenation	Autologous skeletal muscle	Direct injection of autologous mitochondria into the ischemic myocardium	N/A (2017–2027)	Recruiting/ Boston, Massachusetts, United States	NCT02851758	No published results available
Coronary Artery Bypass Grafting (CABG) + left ventricular dysfunction	Autologous skeletal muscle ± MSC exosomes	Intracoronary and intramyocardial injection (during surgery) /1 × 10[7] mitochondria	I/II (2022–2024)	Recruiting/ Tehran, Iran	NCT05669144	Combined exosome co-transplantation strategy /No published results available
Refractory polymyositis/dermatomyositis	Allogeneic umbilical cord MSC (PN-101)	Intravenous infusion	I/IIa (2021–2023)	Completed/ Seoul, South Korea	NCT04976140	Safety and tolerability /No published results available
Pearson marrow-pancreas syndrome	Autologous haematopoietic stem cell-enriched	Autologous stem cell transplantation	I/II (2019–2021)	Completed/ Ramat Gan, Israel	NCT03384420	Completed in paediatric patients/No published results available

#### Challenges and strategies in addressing neurological disorders

Compared with applications in cardiovascular disorders, mitochondrial transplantation for neurological conditions faces several unique challenges. First, the narrow therapeutic window in acute ischaemic stroke is limited to few hours, which poses challenges for autologous mitochondrial transplantation. The ongoing trial NCT04998357 particularly addresses this constraint by integrating bedside muscle biopsy with rapid mitochondrial isolation during standard endovascular reperfusion therapy. Second, exogenously delivered mitochondria may also become DAMPs, with the potential to trigger gliosis and exacerbate neuroinflammatory responses. Third, the long-term survival, integration, and functional persistence remain uncertain, particularly in chronic neurodegenerative diseases, where a single administration is unlikely to provide sustained benefits.

Several strategies may help to overcome these barriers. Cryopreservation could improve clinical feasibility, as placenta-derived mitochondria retain ATP-generating capacity following storage at -80 °C for up to 2 weeks [174]. This may replace the use of autologous mitochondria isolated from the patient’s own healthy tissue, e.g., skeletal muscles. In addition, focused ultrasound combined with microbubbles may be used to open the BBB transiently and reversibly, thereby enhancing parenchymal distribution and tissue integration of intra-arterially delivered mitochondria [175]. Together, these considerations highlight the need for carefully optimised delivery strategies, rigorous safety evaluation, and standardised manufacturing protocols before mitochondria transplantation can be broadly applied to neurological disorders.

## Conclusion and future directions

Mitochondria transfer is a key mechanism of intercellular communication that sustains recipient cell energy homeostasis which may modify the progression of neurological diseases when dysfunctional mitochondria are delivered from diseased cells. Despite growing recognition of its importance, the mechanisms that govern mitochondria transfer in neurological disorders are still not fully understood. Fundamental questions persist regarding what guides the selection of distinct transfer routes, such as TNTs, EVs, or free mitochondria release into the extracellular space, in specific pathological contexts, and what quantitative threshold is required to achieve meaningful functional rescue. Addressing these questions is essential for defining when mitochondria transfer serves as a protective quality control mechanism and when it becomes maladaptive.

Against this backdrop, mitochondrial transplantation has emerged as a potential therapeutic strategy for neurological diseases. However, successful clinical translation hinges on overcoming several interrelated challenges. Delivery approaches require further optimisation to enable BBB crossing, precise regional targeting, and controlled tissue distribution. Equally important is therapeutic durability, including the survival, integration, and long-term functionality of transplanted mitochondria, as well as determining whether repeated dosing is necessary or feasible in chronic neurodegenerative conditions. In parallel, safety and standardisation remain critical considerations, encompassing immunogenicity, neuroinflammatory risk, scalable mitochondrial production, rigorous quality control, and sensitive functional readouts capable of distinguishing true therapeutic benefit from spontaneous recovery or reperfusion-associated effects.

As clinical trials advance and next-generation delivery platforms, such as engineered extracellular vesicles and cell-type-targeted systems including MitoCatch, continue to evolve, mitochondrial therapy is transitioning from experimental proof-of-concept towards a more precise and rational intervention. Ultimately, integrating mechanistic insight with technological innovation will be essential to harness mitochondria transfer not merely as a rescue strategy, but as a controllable and disease-modifying therapeutic modality for neurological disorders.

## Data Availability

No datasets were generated or analysed during the current study.
